# MicroRNA-7: a promising new target in cancer therapy

**DOI:** 10.1186/s12935-015-0259-0

**Published:** 2015-10-29

**Authors:** Juanjuan Zhao, Yijing Tao, Ya Zhou, Nalin Qin, Chao Chen, Dan Tian, Lin Xu

**Affiliations:** Department of Immunology, Zunyi Medical College, Guizhou, 563000 People’s Republic of China; Department of Medical Physics, Zunyi Medical College, Guizhou, 563000 China

**Keywords:** Tumors, MiR-7, Diagnosis, Treatment

## Abstract

The incidence of tumors with life-threatening effects has increased gradually over time; however, the mechanisms involved in tumor development have not been fully elucidated. Recent studies have shown that microRNA-7 (miR-7), which is endogenous non-coding RNA molecules of approximately 23 nucleotides, plays an important role in the occurrence and development of tumors as a key tumor suppressor. Mechanistic evidence showed that miR-7 is closely related to the growth, metastasis, and prognosis of various malignant tumors through regulating different target molecules, which suggest that miR-7 may be a new target for the clinical diagnosis and treatment of various tumors. In this review, we summarize current knowledge of the relationship between miR-7 and tumor development, diagnosis, and treatment.

## Background

MicroRNAs (miRNAs) are small noncoding RNAs that posttranscriptionally regulating target gene expression through base pairing to partially complementary sites to prevent protein accumulation by repressing translation or by inducing mRNA degradation [1, 2]. The primary transcripts of miRNA (pri-miRNA) are cleaved into precursor miRNA (pre-miRNA) by nuclear RNase III Drosha, and further processed to mature miRNAs by cytoplasmic RNase III Dicer [3, 4]. Up to data, the involvement of miRNAs in human cancer has led to extensive research [5–7]. More and more evidences showed that miRNAs were not only dysregulated in almost all types of cancers but also were potential specific signatures for the characterization of poorly differentiated tumors, which might harbor relevant clinical implications. Further studies suggested that miRNAs in cancers could be divided into two types including oncogenic miRNAs (Oncomirs) and tumor suppressor miRNAs. Oncomirs are reported upregulated in cancer cells and contributed to carcinogenesis by inhibiting tumor suppressor genes. Conversely, tumor suppressor miRNAs are documented could prevent cancer development by inhibiting the expression of oncogenes. Experimental data in some animal tumor models showed that silencing oncomirs with miRNA inhibitors or replacing tumor suppressor miRNAs with synthetic miRNA mimics may be valuable strategy for the treatment of solid and hematological malignancies, which suggesting a particularly promising in translational therapy against tumors.

**Fig. 1:**
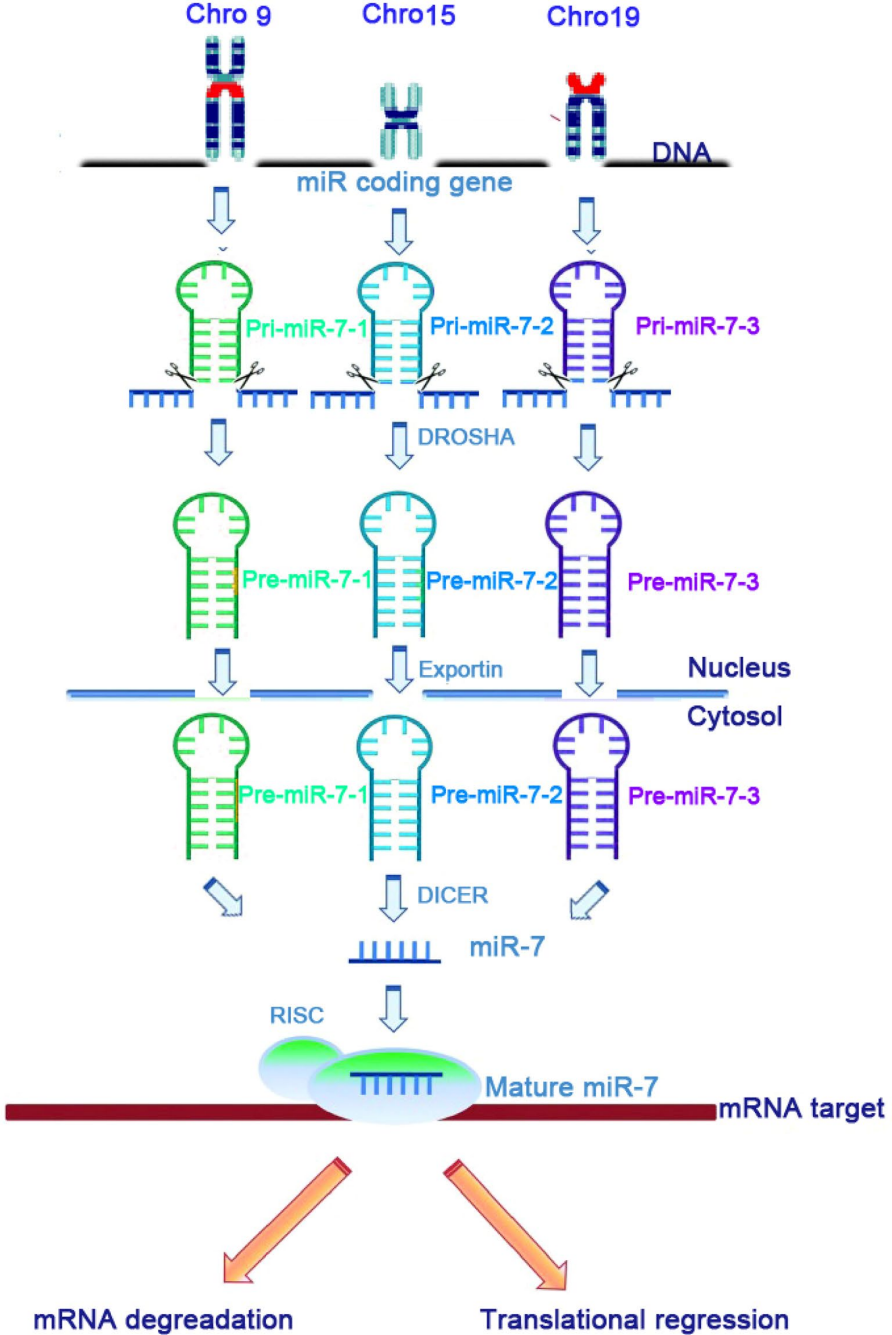
Structure of the human miR-7 hairpin precursors and mature sequences. MiR-7 is transcribed from three different genomic loci on chromosomes 9, 15, 19 into primary miR-7 transcripts (pri-miR-7-1, pre-miR-7-2, pri-miR-7-3 respectively), which are processed into hairpin precursor molecules (pre-miR-7-1, pre-miR-7-2, pre-miR-7-3), and then further into the same mature miR-7 sequences, which are incorporated into the RISC complex and guided to miR-7 target mRNAs to repress their expression

MicroRNA-7 (miR-7) was first identified by Lagos-Quintana in 2001 [8]. In humans, miR-7 is encoded by three different genomic loci 9q21, 15q26, 19q13 respectively. The products of three different DNA sequences (termed as pri-miR-7-1, pri-miR-7-2, and pri-miR-7-3) can be processed into the same mature miR-7 sequence which comprising 23 nucleotides [9]. The mature miR-7 is then incorporated into a RNA-induced silencing complex (RISC), which recognizes target mRNAs through imperfect base pairing with the miRNA and most commonly results in translational inhibition or destabilization of the target mRNA (Fig. 1). Many studies have reported that miR-7 is enriched in a variety of normal tissues and involved in both the development of multiple organs and biological functions of cells [10, 11]. Moreover, some tissues related factors could regulate the expression of miR-7; for example, transcription factor NeuroD/Beta2 can bind to the promoter region of miR-7 and successively regulate its expression [12]. And Human antigen R (HuR) could mediate the binding of Musashi homolog 2 (MSI2) to the conserved terminal loop of pri-miR-7, then modify miR-7 processing in normal cells [13]. Recent evidence showed that miR-7 plays an important role in growth, migration, and invasion of multiple cancers [14–17]; hence, it may be a new promising target for cancer therapies.

## MiR-7 and breast cancer

E-cadherin and Vimentin were reported played crucial roles in epithelial-mesenchymal transition (EMT) process, including monolayer scattering, independent growth, migration, and invasion, of breast cancer cells, which closely related to metastasis of cancer cells [18]. A study of breast cancer tissue specimens revealed a positive correlation between the expression levels of miR-7 and E-cadherin, and a negative correlation between the expression levels of miR-7 and Vimentin [19], indicating miR-7 might be involved in the metastasis of breast cancer. Importantly, the expression level of miR-7 in metastatic breast cancer tissues was significantly lower than that in normal breast tissues. Furthermore, an increase in the expression level of miR-7 could dramatically inhibit the growth and invasiveness of breast cancer cells in animal models, indicating that miR-7 plays an important role in metastasis of breast cancer [19].

Successive studies have demonstrated that miR-7 can negatively regulate a variety of molecules involved in breast cancer cell growth, metastasis, and invasion. P21-activated kinase 1 (PAK1) is expressed at high levels in many human cancer tissues and is closely related to mitosis of tumor cells, regeneration of the cytoskeleton structure, cell migration and apoptosis [20]. Reddy et al. showed that miR-7 binds to a complementary site in the 3′ untranslated region (UTR) of the *PAK1* mRNA to inhibit its expression and downregulate the kinase activity of the protein, resulting in the inhibition of breast cancer growth [21]. In addition, Webster et al. reported that miR-7 inhibits epidermal growth factor receptor (EGFR) expression and the protein kinase B signal transmission pathway, thereby regulating the growth and metastasis of breast cancer cells [22]. MiR-7 can also inhibit the invasion and metastasis of breast cancer cells by negatively regulating focal adhesion kinase (FAK), an important signaling factor that controls cell proliferation and locomotor activity [19]. In line with these data, the expression level of FAK is increased in metastatic tumors with poor prognosis [23]. In additions, miR-7 also can inhibit brain metastasis of breast cancer and the self-renewal capacity of breast cancer stem-like cells by regulating the expression of Krüppel-like factor4 (KLF4) [24].

The underlying mechanism through which miR-7 regulates breast cancer cell growth and metastasis is complex, it dose not involve only the above mentioned molecular targets and signal transmission pathways but also some transcription factors and DNA damage repair processes. For example, Li et al. demonstrated that miR-7 can reactivate the Ras association domain family 1A (Raf1A) and tumor suppressors claudin-6 by targeting the gene encoding homeobox B3 (HoxB3), therefore inhibit the growth and colony formation capacity of breast cancer cells [25]. In addition, Yu et al. showed that miR-7 binds to the 3′UTR of the mRNA encoding SET domain-containing (lysine methyltransferase) 8 (SET8) to downregulate its expression in breast cancer cells [26]. In higher eukaryotes, SET8 is involved in gene transcription and the cell cycle progression of breast cancer cells, and is also related to a variety of biological processes, including DNA damage and repair [27]. Moreover, MiR-7 can reduce the monomethylation of histone H4 lysine 20 by targeting SET8, then inhibiting the occurrence, development, and invasion of breast cancer [26]. In addition, some studies reported that miR-7 accelerates spontaneous DNA damage and sensitizes breast cancer cells to this type of damage. Taken together, these findings indicate that miR-7 can regulate the development and progression of breast cancer through multiple targets or pathways; hence, miR-7 based breast cancer gene therapy is expected to become an important area of related research.

The current understanding of the mechanism of regulating miR-7 expression in breast cancer cells still is limited. MiR-7 expression is upregulated by the transcription factor HoxD10. When the level of HoxD10 is decreased, the expression level of miR-7 is also decreased. Successively, the expression level of its target molecule PAK1 is increased significantly, the growth and invasiveness of breast cancer cells is enhanced [21]. Due to the complexity of the mechanisms of regulation of miRNAs, additional analyses on off factors that affect miR-7 expression in breast cancer are required.

## MiR-7 and lung cancer

MiR-7 plays an important role in suppressing the migration, colony formation, and cell-cycle progression of lung cancer cells. Reducing the amount of miR-7 can promote the growth and metastasis of human lung carcinoma A549 and H1299 cells significantly [28]. Furthermore, increasing the level of miR-7 can reduce the levels of cell-cycle-associated proteins and inhibit the proliferation of human lung cancer cells, suggesting that miR-7 is closely related to development of lung cancer. It is well known that epidermal growth factor receptor (EGFR) is critical factor for the growth and metastasis of human lung cancer cells [29, 30]. Mechanistic evidence showed that miR-7 can inhibit the proliferation of lung cancer cells through regulating the expression of EGFR by binding to the 3′UTR region of EGFR mRNA [28]. In addition, Xiong et al. showed that miR-7 downregulated the expression of the gene encoding BCL-2, thereby inhibiting the proliferation and promoting the apoptosis of lung adenocarcinoma A549 cells [31]. Other studies also reported that miR-7 targets and downregulates proteasome activator 28 subunit γ, which contributes to the carcinogenesis of non-small cell lung cancer, and inhibits apoptosis and accelerates the cell cycle in lung cancer cells [32]. Moreover, Zhou et al. showed that the regulatory effect of miR-7 on the growth of human lung cancer cells may be associated with the downregulated expression of Solute Carrier Family 7 (Amino Acid Transporter Light Chain, L System), Member 5 (SLC7A5), which was a member of the l-type amino acid transporter (LAT) family and was involved in the growth of cancer cells [33–35]. In contrast to these literatures, other studies showed that inhibition of miR-7 could reduce the growth of lung cancer cell line A549 cells [36]. Based on these contradictory data, we propose that this might reflect the fact that miR-7 plays a crucial role in lung cancer cell growth, invasion, and metastasis, although the mechanisms involved in regulating these processes may be complex, which was closely related to the cellular context, growth conditions and specific targeted genes.

A number of studies have shown that miR-7 also is involved in the signal transduction and regulatory processes of other membrane-bound proteins in lung cancer cells, such as the pattern-recognition molecules Toll-like receptor 9 (TLR9). Our recent study showed that miR-7 may negatively regulate the phosphoinositide 3-kinase (PI3K)/AKT signaling pathway by acting on PI3Kregulatory subunit 3, thereby blocking the TLR9 signal transduction process and inhibiting cancer cell growth and metastasis [37]. Furthermore, activation of TLR9 signaling can inhibit the cleavage of the endogenous miR-7 precursor in lung cancer cells by inducing the expression of human antigen R (HuR), therefore reduce the level of mature miR-7 and accelerate the growth and metastatic progression of the cells [38].

The treatment of lung cancer could lead to an acquired resistance to EGFR tyrosine kinase inhibitors (TKIs). However, in about 50 % of patients with TKIs resistance, this resistance is caused by a T790M mutation in exon 20 of the *EGFR* gene [39]. Interestingly, Rai et al. showed that expression of exogenous miR-7 in nude mice bearing RPC-9 and H1975 lung cancer cells, which have a high tolerance to TKIs, inhibited the growth of tumors, suggesting that miR-7, as a tumor suppressor miRNA, may be an important target for lung cancer gene therapy [40].

## MiR-7 and brain cancer

MiR-7 is enriched in the brain and regulates neuronal differentiation, maturation, and survival [41].There is increasing evidence that miR-7 is a robust suppressor of the growth, metastasis, and invasion of glioblastoma; for example, Liu et al. showed that miR-7 can suppress glioblastomagenesis by regulating the PI3K/AKT and Raf/mitogen-activated protein kinase/extracellular signal-regulated kinase (ERK) pathways, which are downstream signaling pathways of the EGFR [42]. Furthermore, miR-7 may regulate brain tumorigenesis by targeting oncogenic proteins and critical factors such as c-KIT, Y box binding protein 1, AKT2, cell division control protein 42, cyclin-dependent kinase 6, and transforming growth factor β2 (TGF-β2), which can also affect the expression of miR-7 in a feedback loop [43]. Similar to the anti-angiogenic drug sunitinib, miR-7 can reduce vascularization in the chick chorioallantoic membrane assay. Furthermore, local application of miR-7 to neuroblastoma model mice harboring human glioblastoma xenografts reduces angiogenesis and tumor cell proliferation by targeting the 3′UTR of the *FAK* mRNA [44]. In addition, miR-7 also can inhibit glioblastoma cell invasion by downregulating the expression levels of p-ERK1/2, p-AKT, Raf-1, and cyclin D1 [42]. There also are some reports suggesting that brain-enriched ciRS-7, a circular RNA containing 70 conserved miR-7 target sites, can downregulate the expression of miR-7 and promote the development of neuroblastomas and astrocytomas [45]. Conversely, miR-671 can release the expression of miR-7 by binding to ciRS-7 [46]. Overall, these findings suggest that miR-7 may be a potential therapeutic target to suppress brain tumorigenesis, metastasis, and invasion.

## MiR-7 and other tumors

Recently, a large number of studies have demonstrated that miR-7 is related to liver cancer [47], colorectal cancer [48], gastric tumor [49], and ovarian cancer [50]. Using immunohistochemistry, real-time PCR, and luciferase reporter genes assay, Needhamsen et al. showed that the human gene encoding paired box 6 (PAX6) is an putative target of miR-7 [51]. PAX6 is a highly conserved transcription factor that is expressed in colorectal cancer cells and plays an important regulatory role in the development of colorectal cancer [52]. Overexpression of PAX6 in human colonic adenocarcinoma SW480 cells activates the ERK/PI3K signaling pathway and upregulates the expression levels of MMP2 and MMP9, resulting in the enhancement of cancer cell growth, proliferation, and metastatic capacity. By contrast, miR-7 reduces PAX6 expression in colorectal cancer cell lines and inhibits the corresponding signaling through the ERK/PI3K pathway, resulting in downregulated growth, proliferation, and metastasis of these cells significantly [53]. Meanwhile, in vivo experiments revealed that miR-7 could bind to the 3′ UTR of the mRNA encoding Yin Yang 1, which was a multifunctional transcription factor and was reported contributed to growth of cancer cells, and inhibit its expression, thereby attenuated colorectal cancer cell growth and invasion [54].

Fang et al. showed that miR-7 arrests hepatocellular carcinoma cells in the G0/G1 phase and negatively regulates the PI3K/AKT pathway by targeting AKT, mammalian target of rapamycin, and ribosomal protein S6 kinase, which are key signaling molecules involved in the promotion of liver cancer cell growth and metastasis [55]. MiR-7 may also play an important role in discriminating between benign and malignant cells in B-chronic lymphocytic leukemia [56]. In addition, Zhao et al. showed that miR-7 binds to the 3′ UTR of the mRNA encoding insulin-like growth factor 1 receptor and induces cadherin expression and reversal of the epithelial-mesenchymal transition of gastric cancer cells, successively inhibited gastric cancer metastasis [57]. A number of other types of cancer are also reported closely related to miR-7 (summarized in Table 1), suggesting that this miRNA may be a novel clinical target for the treatment of a variety of tumors.

**Table 1:** The targets of miR-7 in different tumor types

Tumor type	Target site(s)	Reference(s)	Role of target site
Breast cancer	FAK, PAK1, EGFR, KLF4, HoxB3, SET8	[19, 21, 22, 24–26]	Suppressor
Lung cancer	PA28γ, BCL-2, EGFR, SLC7A5, HuR	[32, 35, 38 39]	Suppressor
Hepatocellular carcinoma	CCNE1, PI3KCD, mTOR, p70S6 K, GSK-3B	[48, 54]	Suppressor
Glioma	EGFR, C-KIT, FAK, ERK, ciRS-7	[42–44, 46]	Suppressor
Colorectal cancer	XRCC2, PAX6, YY1	[49, 52, 53]	Suppressor
Gastric cancer	EGFR, IGF1R	[42, 56]	Suppressor
Acute myelocytic leukemia	TET2	[58]	Suppressor
Urothelium carcinoma	FGFR3	[59]	Suppressor
Cervical cancer	XIAP	[60]	Suppressor
Renal carcinoma	None reported	[61]	Oncogene
Melanoma	IRS-2	[62]	Suppressor
Ovarian cancer	EGFR, ERK	[50]	Suppressor
Oral cancer	RECK	[63]	Oncogene
Neuroblastoma	4-HPR, EGCG	[64]	Suppressor
Pancreatic carcinoma	MAPK	[65]	suppressor
Pleural mesothelioma	None reported	[66]	Suppressor
Schwannoma tumors	EGFR, ACK1, PAK1	[67]	Suppressor
Tongue squamous carcinoma	IGF1R	[68]	Suppressor

*FAK* focal adhesion kinase, *PAK1* p21 activated kinase 1, *EGFR* epidermal growth factor receptor, *KLF4* Krüppel like factor 4, *HoxB3* homeobox B3, *SET8* SET domain-containing (lysine methyltransferase) 8, *PA28γ* proteasome activator 28 subunit γ, *HuR* human antigen R, *PI3KCD* phosphoinositide 3-kinase catalytic subunit delta, *mTOR* mammalian target of rapamycin, *p70S6* *K* ribosomal protein S6 kinase, *GSK*-*3β* glycogen synthase kinase 3, *ciRS*-*7* circular RNA sponge for miR-7, *XRCC2* X-ray repair complementing defective repair in Chinese hamster cells 2, *PAX6* paired box 6, *YY1* Yin Yang 1, *IGF1R* insulin-like growth factor 1 receptor, *TET2* tet methylcytosine dioxygenase 2, *FGFR3* fibroblast growth factor receptor 3, *XIAP* X-linked inhibitor of apoptosis protein, *IRS*-*2* insulin receptor substrate 2, *RECK* reversion-inducing cysteine-rich protein with Kazal motifs, *4*-*HPR* *N*-(4-hydroxyphenyl) retinamide, *EGCG* (-)-epigallocatechin-3-gallate, *MAPK* mitogen-activated protein kinase, *ACK1* associated cdc42 kinase 1

## MiR-7 and tumor diagnosis, therapy

Multiple studies have suggested that miR-7 can be used as a potential diagnostic marker of cancer. For example, the expression level of miR-7 is significantly lower in lung cancer tissues than normal lung tissues, and downregulation of miR-7 expression is associated with lung cancer metastasis [32]. Kitano et al. showed that miR-7 expression level predicts thyroid cancer prognosis with an accuracy of approximately 76 % [69]. Other groups used non-invasive detection methods and found that the serum level of miR-7 is significantly lower in patients with colorectal cancer than healthy controls [70]. Moreover, fecal expression levels of miR-7 was remarkable difference between colorectal cancer patients and healthy controls, suggesting that non-invasive detection of miR-7 can be used for the auxiliary diagnosis of colorectal cancer [71]. In addition, other studies have suggested that miR-7 may be an important clinical predictor of recurrence. For example, Duncavage et al. showed that the expression level of miR-7 in relapsed lung cancer patients is significantly lower than that in healthy individuals, suggesting a relationship between reduced expression of miR-7 and lung cancer recurrence [72].

To the therapy against cancer, the related progression still is limited but promising. Overexpression of miR-7 using a eukaryotic expression vector can inhibit the growth of TKI-resistant lung cancer cell significantly in vivo. Tazawa et al. constructed an oncolytic adenovirus that upregulated miR-7 expression and induced apoptosis of human tumor cells through the activation of the transcription factor E2F1/miR-7/EGFR pathway, suggesting a potential new method of inducing tumor cell death by controlling miR-7 levels [73]. In addition, Lee et al. reported that overexpression of miR-7 increases the radiosensitivity of cancer cells, suggesting that this miRNA may have a positive effect on radiation therapy of tumors [74]; however, further analyses are required to clarify the relationship between tumor diagnosis/treatment and the expression levels of miR-7 in different tissues and organs. In all, the development of miR-7-based therapeutic strategy, including the application of efficient synthetic systems for miR-7 delivery, remains to be fully investigated in future.

## Conclusion

In this review, we have tried to discuss miR-7 as a promising target in cancers. Although substantial progress has been made in the mechanisms by which miR-7 controls tumor cell growth, proliferation, invasion, and metastasis, however, a number of issues require further investigation, such as the precise regulatory mechanisms of miR-7, the relationships between its target genes, and the ways in which targeting miR-7 can be used to develop agents that have desirable effects on distinct type of cancers, which will provide robust and valuable information related to the clinical diagnosis, treatment, and prognoses of various cancers.
